# Animal Models of Parkinson's Disease 2012

**DOI:** 10.1155/2012/729428

**Published:** 2012-09-16

**Authors:** Yuzuru Imai, Katerina Venderova, Kah-Leong Lim

**Affiliations:** ^1^Department of Neuroscience for Neurodegenerative Disorders, Juntendo University Graduate School of Medicine, Tokyo 113-8421, Japan; ^2^Department of Physiology and Pharmacology, Thomas J. Long School of Pharmacy and Health Sciences, University of the Pacific, Stockton, CA 95211, USA; ^3^Department of Physiology, National University of Singapore, Block MD9, 2 Medical Drive, Singapore 117597

Parkinson's disease (PD) is a prevalent movement disorder, which is characterized by age-dependent degeneration of dopaminergic neurons in the midbrain. Recent advances in PD study have suggested that multiple nervous systems are affected as well as the nigrostriatal system, involving decline of cognitive function, sleep disturbance, mood change, and dysfunction of the autonomic nervous system. Multifactorial causes of PD are hypothesized. Neurotoxins including artificial compounds, pesticides, heavy metals as well as dopamine itself have been proposed to be environmental risk factors of PD. Recent genome-wide genetic and mutational studies have revealed various genetic risk factors and SNPs while microglial activation in the affected regions have emerged to be involved in the disease development as a local microenvironmental factor. One of the pathological hallmarks of PD is the appearance of ubiquitin-positive cytoplasmic inclusions called Lewy bodies (LBs) in the affected regions. A presynaptic protein *α*-synuclein, which is the first identified monogenic PD gene product, is a major component of LBs. Understanding of the neuropathological mechanism underlying the formation of LBs is a key aspect of PD study. Newly identified monogenic PD gene products, leucine-rich repeat kinase 2 (LRRK2), ATPase type 13A2, and Vps35 are implicated in vesicle transport, endosomal—autophagic and lysosomal pathways. Dysregulation of these gene products might be involved in abnormal protein turnover associated with the LB formation. 

Dysfunction of mitochondrial pathway and oxidative stress are also a focus based on the findings that PD-related neurotoxins such as 1-methyl-4-phenyl-1,2,3,6-tetrahydropyridine (MPTP), 6-hydroxydopamine (6-OHDA), and rotenone could interfere with mitochondrial functions and that monogenic PD gene products DJ-1, PINK1, and Parkin are involved in mitochondrial maintenance and redox regulation.

Autologous cell transplantation is fast becoming a reality now that technology for induced pluripotent stem (iPS) and induced dopaminergic (iDA) cells from fibroblasts achieves improvement on a daily basis [1]. At the same time, the methodology of gene therapy is also enhanced with the use of new DNA delivery tools. A wide variety of PD animal models contribute to the understanding of neuropathological mechanisms described above and the development of therapeutic approaches as an alternative to humans, although none of them have fully recapitulated the symptoms and pathology of PD so far. 

This special issue is composed of an excellent review and 4 distinguished original articles that summarize the most recent progresses and ideas obtained from animal models in the specific fields, while reporting a new sensitive assay for PD models and a potential therapeutic approach.

The review paper briefly outlines newly developed rat models for *α-synuclein*- or *LRRK2-*linked PD. Aggregation of *α*-synuclein, which is accelerated by pathogenic mutations and phosphorylation at Ser129, is believed to lead to LB formation. Rat models introduced with these forms of *α*-synuclein have revealed *in vivo* effects of the aggregation-prone *α*-synuclein. Genome-wide association studies of sporadic PD have identified *LRRK2* and *α-synuclein* as risk loci, suggesting that these two genes are closely involved in the fundamental neuropathology of PD [2, 3]. Rat models for LRRK2 are also discussed as tools for potential therapeutic research.

Neurotoxicity of glutamate, which is the major excitatory neurotransmitter of neurons, is known to occur after brain injury and spinal cord injury. G. Bustos et al. have reported that activation of NMDA receptor upon glutamate release evokes BDNF expression in the early presymptomatic response of the *substantia nigra* in PD [4]. A current study by E. Riquelme et al. addresses the possibility of trkB involvement in this pathway.

Near-infrared light (NIr) treatment has a neuroprotective effect on dopaminergic neurons in the *substantia nigra* exposed to MPTP, presumably through the regulation of the mitochondrial activity and redox status [5, 6]. V. E. Shaw et al. examine an effect of NIr treatment on the subthalamic region in MPTP-treated mice, by estimating abnormally activated Fos-positive cells.

*Drosophila melanogaster* is a useful model system for human disease study as ~75% of disease-associated genes are conserved between humans and *Drosophila*. Although the orthologue of *α*-synuclein gene is not found in the *Drosophila* genome, transgenic expression of *α*-synuclein causes progressive motor defects, which is usually evaluated by climbing ability in a test for negative geotaxis [7]. R. Shaltiel-Karyo et al. report here that defects in courtship-associated behavior is detectable at a much earlier stage of the *α*-synuclein model fly. Their assay could be an alternative to detect impairment of the coordinated motor activity in PD model files.

Minocycline (MC), which has anti-inflammatory and anti-oxidative properties, is a promising drug for neurodegenerative disease; however this drug has deleterious effects on some neurodegenerative models [8, 9]. To understand and control the diverse actions of MC, A. A. Inamdar et al. find that genetic factors modulate the effects of MC using a *Drosophila* model, a powerful tool to elucidate the interactions between drugs and genes.

*Yuzuru Imai* *Yuzuru Imai* *Katerina Venderova* *Katerina Venderova* *Kah-Leong Lim* *Kah-Leong Lim*
